# A Review of Discovery Profiling of PIWI-Interacting RNAs and Their Diverse Functions in Metazoans

**DOI:** 10.3390/ijms222011166

**Published:** 2021-10-16

**Authors:** Songqian Huang, Kazutoshi Yoshitake, Shuichi Asakawa

**Affiliations:** Department of Aquatic Bioscience, Graduate School of Agricultural and Life Sciences, The University of Tokyo, Bunkyo, Tokyo 113-8657, Japan; akyoshita@g.ecc.u-tokyo.ac.jp

**Keywords:** PIWI-interacting RNA, piRNA discovery, piRNA function, transposable elements, sRNA-seq, metazoans

## Abstract

PIWI-interacting RNAs (piRNAs) are a class of small non-coding RNAs (sncRNAs) that perform crucial biological functions in metazoans and defend against transposable elements (TEs) in germ lines. Recently, ubiquitously expressed piRNAs were discovered in soma and germ lines using small RNA sequencing (sRNA-seq) in humans and animals, providing new insights into the diverse functions of piRNAs. However, the role of piRNAs has not yet been fully elucidated, and sRNA-seq studies continue to reveal different piRNA activities in the genome. In this review, we summarize a set of simplified processes for piRNA analysis in order to provide a useful guide for researchers to perform piRNA research suitable for their study objectives. These processes can help expand the functional research on piRNAs from previously reported sRNA-seq results in metazoans. Ubiquitously expressed piRNAs have been discovered in the soma and germ lines in Annelida, Cnidaria, Echinodermata, Crustacea, Arthropoda, and Mollusca, but they are limited to germ lines in Chordata. The roles of piRNAs in TE silencing, gene expression regulation, epigenetic regulation, embryonic development, immune response, and associated diseases will continue to be discovered via sRNA-seq.

## 1. Introduction

Small non-coding RNAs (sncRNAs) engage in gene regulation at the transcriptional and post-transcriptional levels and are classified as microRNAs (miRNAs), endogenous small interfering RNAs (endo-siRNAs), and PIWI-interacting RNAs (piRNAs) based on their size and Argonaute partner in biogenesis [1]. piRNAs form the largest and most heterogeneous class of sncRNAs because they lack conserved structural motifs and sequence homology across species [2,3]. Studies on piRNAs have attracted significant attention from researchers in the last decade.

Double-stranded small RNA derived from the suppressor of the Stellate locus on the Y chromosome was first discovered in the *Drosophila melanogaster* germ line [4]. Repeat-associated small interfering RNAs (rasiRNAs) were first identified in *Drosophila* germ lines [4,5,6], and were later termed piRNA subspecies as they were found to interact with PIWI proteins [7]. Remarkable progress has been made in understanding piRNA biogenesis and function, especially in *Drosophila*, *Caenorhabditis elegans*, mice, and humans [8,9,10,11,12,13,14,15,16,17,18,19]. Next-generation sequencing (NGS) has been widely used for high-throughput characterization of sncRNAs. Increasingly, piRNAs have been discovered in the soma and germ lines of non-model organisms, including Platyhelminthes [20], Annelida [21], Cnidaria [22,23,24,25], Echinodermata [26], Mollusca [27], Crustacea [28], Arthropoda [29], Reptilia [30], and Mammals [31].

In the last decade, several studies have attempted to elucidate the biogenesis of piRNAs [32,33,34,35,36,37]. Two models of the piRNA biogenesis pathway have been demonstrated in various animals: the primary piRNA biogenesis pathway and the amplification loop or ping-pong cycle [32]. In the primary piRNA biogenesis pathway, long piRNA precursors are transcribed from piRNA clusters, cleaved and modified by complex factors in the cytoplasm, and then transported into the nucleus in complex with PIWI proteins [38]. piRNAs generated by the primary pathway may play a role in regulating gene expression [32]. Secondary piRNAs are formed in an amplification mechanism (termed the ping-pong amplification loop) to specifically enhance piRNA sequences [35,36].

The PIWI–piRNA pathway effectively suppresses transposable element (TE) activity in order to safeguard the genome from detrimental insertion mutagenesis [39]. Recent findings show that the PIWI–piRNA pathway also plays a vital role in somatic cells [40,41] and various cancer cells [42,43,44,45]. The present review aims to provide guidelines for piRNA discovery in future studies. We discuss the discovery profiling of piRNAs in model and non-model organisms using small RNA sequencing (sRNA-seq) and provide an overview of piRNA functions in animals. In addition, we rediscovered ubiquitously expressed piRNAs in the soma and germ lines of invertebrates from previously overlooked sRNA-seq data. Overall, discovering piRNAs can assist researchers in analyze their functions in non-model organisms.

## 2. Identification of piRNA

### 2.1. Discovery Workflow

**Figure 1:**
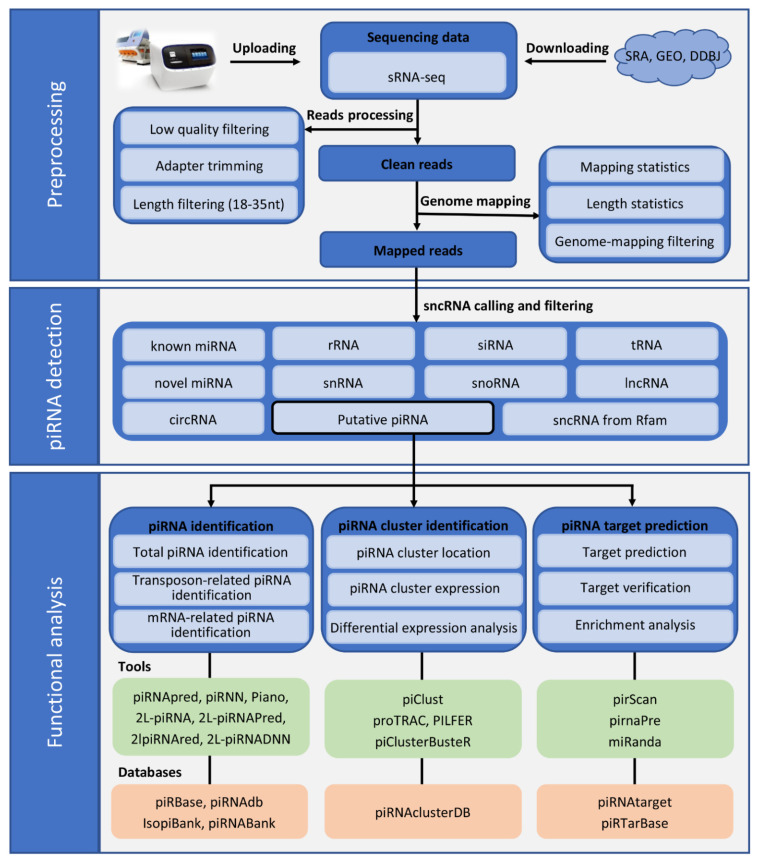
Overview of common pipeline for PIWI-interacting RNA (piRNA) discovery and functional analysis in metazoans. The raw data of small RNA sequencing (sRNA-seq) were trimmed using adapters, quality control was performed, and the data were subsequently filtered by read lengths. Generally, microRNAs (miRNAs) and small interfering RNAs (siRNAs) are 20–25 nt in length, transfer RNAs (tRNAs) are typically ~80 nt in length, and small nuclear RNAs (snRNAs) and circular RNAs (circRNAs) are more than 100 nt in length, whereas piRNAs normally have lengths of 24–31 nt. However, species-specific piRNAs of 21 nt with a 5′ uridine (21U-RNAs) binding to p53-responsive gene 1 (PRG-1) and 22 nt piRNAs with a 5′ guanosine (22G-RNAs) loaded onto worm-specific Argonautes (WAGOs) were detected in *C. elegans* [50,51,52]. In the preprocessing step, the potential piRNAs and piRNA isoforms with a length of 18–35 nt were preserved for subsequent known RNA mapping and filtration. The remaining putative piRNA reads were processed for piRNA analysis using multiple tools and databases.

Identifying piRNAs from sRNA-seq is imperative for further functional analysis (Figure 1). Samples from tissues or cells were prepared for sRNA-seq to identify the piRNA molecules. Raw data from high-throughput sequencing required trimming adapters and quality control processes, such as filtration of low-quality reads, poly(A) reads, or length, to obtain clean reads. Moreover, the clean small RNAs were aligned with genome sequences and well-known RNA databases for the filtration of infectant reads and known RNA molecules, such as ribosomal RNAs (rRNAs), miRNAs, and small interfering RNAs (siRNAs). Generally, piRNA sequences are represented by ncRNA fragments, while some piRNA databases contain a subset of sequences that correspond to piRNA-sized fragments of ncRNAs (rRNAs, transfer RNAs (tRNAs), small nuclear RNAs (snRNAs), and small nucleolar RNAs (snoRNAs)) and intermediates of miRNA biogenesis, which strongly affect the estimation of piRNA expression outside mammalian gonads [46,47]. Therefore, all known ncRNA fragments should be thoroughly filtered out when analyzing somatic piRNAs in mammals. Finally, the putative reads were processed experimentally or using bioinformatics tools to identify the piRNA molecules.

Crosslinking immunoprecipitation sequencing (CLIP-seq) and RNA immunoprecipitation sequencing (RIP-seq) are commonly used to detect piRNAs with the coprecipitation of PIWI/Argonaute. The experimental method is powerful, allowing unambiguous classification of precipitated small RNAs and elucidation of the functions of various PIWI or Argonaute proteins, but with the disadvantages of being time-consuming and expensive [48,49]. Therefore, specialized bioinformatics tools for piRNA identification and processing on a large scale are required.

### 2.2. Processing of piRNAs

The absence of many conserved structural and sequence characteristics makes it difficult to identify piRNAs using computational methods. An exception to this is their preference for a uridine nucleotide at the first position (1U) from 5 terminal [53]. A hallmark characteristic of piRNA sequences is their appearance in genome clusters ranging from 1 to >100 kb in length, with both monodirectional and bidirectional encoding clusters [54]. Moreover, secondary piRNAs show a strong bias for adenine at position 10 (10A), complementing the 1U bias of primary piRNAs [55].

In the last decade, scientists have developed various computational methods to identify piRNAs from sRNA-seq. These methods can be classified into two categories: linear classification algorithms to predict individual piRNAs and clustering approaches to predict clustered piRNAs [3]. One aim in identifying piRNAs is to summarize the general features of known piRNAs from model organisms with complete genome sequences and use them to predict novel piRNAs [2]. Several methods have been developed to predict individual piRNAs based on their type. For example, Pinao [56], a genetic algorithm-based weighted ensemble (GA-WE) [57], and accurate piRNA prediction [58] have been used for transposon-related piRNA prediction, and two-layer integrated programs for identifying piRNAs (2L-piRNA) [59], such as 2L-piRNAPred [60], 2lpiRNApred [61], and 2L-piRNADNN [62], have been developed for mRNA-related piRNA prediction, while piRNAPredictor [2], PiRPred [3], piRNAdetect [63], IpiRId [64], piRNN [65], and piRNApred [66] have been employed for total piRNA prediction. miRanda [17], pirnaPre [67], and pirScan [18] have been used for piRNA target prediction, and three algorithms have been proposed for predicting piRNA clusters from sRNA-seq data: proTRAC [54], piClust [68], and PILFER [69]. In addition, multiple integrated platforms, such as sRNAtools [70] and Workflow for piRNAs and Beyond (WIND) [71], have been recently developed for piRNA annotation and downstream analysis from raw data to plots and statistics by sRNA-seq. The performances of most of these piRNA prediction tools have been reviewed by Liu et al. [46].

Multiple piRNA-dedicated databases have been built for piRNA annotation and downstream analysis. These can be divided into different categories according to their functions: piRNABank [72], piRBase [73], and piRNAdb for comprehensive annotation; IsopiRBank [74] for piRNA isoform identification; piRNAclusterDB [75] for piRNA cluster annotation; piRNAtarget [76] and piRTarBase [77] for piRNA-mediated target prediction; and piRDisease [78] and piRPheno [79] for piRNA-related disease analysis. However, most piRNA databases have been generated from model organisms, such as *C. elegans*, *Drosophila*, mice, and humans, which limits their use in non-model organisms.

### 2.3. Validation of piRNA

Northern blotting, in situ hybridization, and quantitative reverse transcription-polymerase chain reaction (qRT-PCR) are the three main approaches for the experimental validation of piRNAs. These methods have low throughput and cannot validate hundreds of piRNAs and their isoforms detected by sRNA-seq. Sequencing of PIWI-precipitated small RNAs is usually used to detect piRNAs directly. However, sRNA-seq of cell lines or tissues before and after the knockdown or knockout of piRNA biogenesis pathway genes can be used to assess the biosynthesis of the predicted piRNAs, as the biogenesis of real piRNAs can be expected to be affected. No piRNAs were detected in zebrafish PIWI (ZIWI) mutant testes in zebrafish [80] or in PIWI mutant fat bodies in *Drosophila* [81].

High-throughput CLIP-seq is another method employed not only for the validation of putative piRNAs but also to verify their activity [82,83]. Overlaps between CLIP-seq tags for putative piRNAs and microprocessor complex subunits or PIWI proteins provide evidence for interactions between the putative piRNAs and the microprocessor or RNA-induced silencing complexes (RISCs) [84]. To determine piRNA targets, CLIP-seq and RIP-seq can identify thousands of transcripts associated with PIWI proteins; however, it is difficult to infer the target of a specific piRNA using these methods [19]. Bioinformatics can be used to first predict the targets of a specific piRNA, but additional approaches are required to validate the predicted binding sites in vivo, such as the dual-luciferase reporter assay with co-transfected piRNA expression vector and wild-type and mutated forms of the predicted 3′ untranslated region (UTR) reporter vector [85]. The interaction between piRNA precursors and intermediate biogenesis factors has also been verified by CLIP-seq [86,87]. Crosslinking, ligation, and sequencing of hybrids (CLASH) has been used to identify small RNAs and candidate target RNA binding sites [88], thus providing direct evidence of piRNA-mediated gene regulation in RISC. CLASH was utilized to study the binding sites between piRNAs and their potential target mRNAs in *C. elegans* [89].

Periodate-mediated oxidation has been used to yield clean piRNA sequences during sRNA-seq processing [21,24,29,31]. The chemical structures of piRNAs were confirmed using this method, followed by β-elimination reactions [90]. It was reported that almost all the piRNAs tested were resistant to periodate treatment, indicating a modified 2′ or 3′ hydroxyl group at the 3′ terminal nucleotides of piRNA, which is methylated by the small RNA methyltransferase HUA ENHANCER1 (HEN1) [91,92].

## 3. Discovery of piRNAs by sRNA-seq

**Figure 2:**
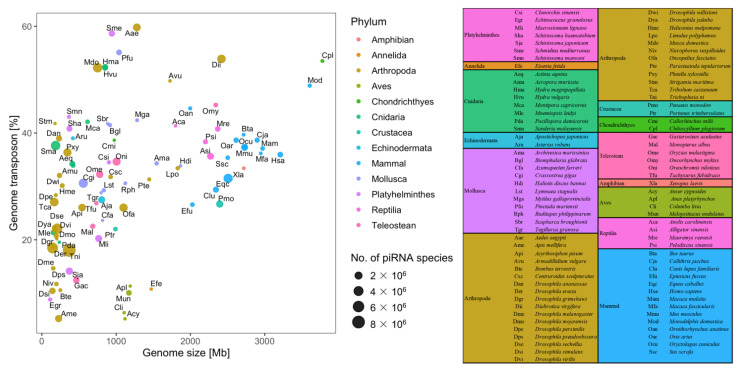
Discovery of piRNAs in metazoans. In the same taxa, the proportion of TEs increases with the genome size, whereas the number of piRNA species does not increase with the size of the genome or the proportion of TEs.

Most of the information on piRNAs is obtained from model organisms such as *Drosophila*; however, continual progress is being made with other organisms belonging to Cnidaria, Mollusca, and Chordata (Teleostean, Amphibian, Reptilia, Aves, and Mammal) (Figure 2). We acquired approximately 1424 sRNA-seq datasets for 114 animal species from public databases for piRNA identification and characterization in invertebrates and vertebrates (Table 1; Supplementary Table S1), including species with and without existing piRNA information. In the same taxa, the proportion of TEs increases with genome size [93], whereas the number of piRNA species does not increase with the size of the genome or the proportion of TEs (Figure 2). piRNAs were not detected in Protozoa but were detected in *C. elegans* and *Halichondria panicea*, belonging to Nematoda and Porifera [14,21]. In Platyhelminthes, piRNAs were detected in planarians (*Schmidtea mediterranea*) [20] but were absent in flukes and tapeworms [94]. Ubiquitously expressed piRNAs were discovered in the soma and germ lines of Annelida, Cnidaria, Echinodermata, Crustacea, Arthropoda, and Mollusca. piRNA expression underwent tremendous changes in the Chordata. They were mostly expressed in early embryos, mammalian testes, and ovaries of *Macaca fascicularis* and *Oryctolagus cuniculus* [31]. piRNAs were also found to exist outside the germ line, particularly in the nervous system of *Aplysia* species [95] and the liver of the bamboo shark (*Chiloscyllium plagiosum*) [96], suggesting much broader roles than previously understood. The somatic piRNA pathway plays a minor role in *Drosophila*, whereas in other Arthropoda somatic piRNAs are more abundant and diversified [29]. The presence of piRNAs in most lower animal species suggests that their last common ancestor had pathways active in both the soma and germ line, and several species in Chordata lost their activity in all but gonadal tissues.

## 4. Diverse Functions of piRNAs

**Figure 3:**
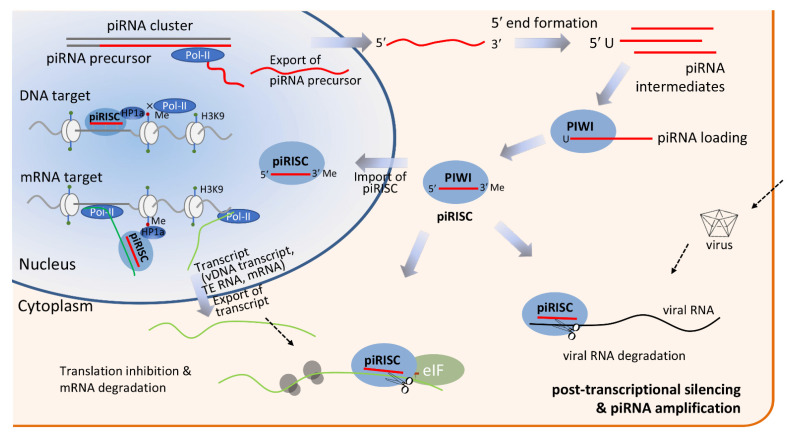
piRNA biogenesis and its functional roles in metazoans. In most cases, the piRNA pathway begins with transcription of piRNA clusters, which is mediated by RNA polymerase II (pol II), to generate the respective precursor piRNA (pre-piRNA) transcripts in the nucleus and drive them to cytoplasm where primary and second piRNA biogenesis takes place. The primary transcripts of piRNA clusters are shortened into piRNA intermediates and subsequently loaded onto PIWI proteins and trimmed from the 3′ end to the size of mature piRNAs and then 2′-O-methylated. The mature piRNAs interact with PIWI proteins to form piRISC, which serves various functions in the nucleus and cytoplasm. piRISC is translocated to the nucleus and targets the nascent transcripts through sequence complementarity. Upon binding, PIWI recruits the epigenetic modifier heterochromatin protein 1 (HP1a) and histone methyltransferase (HMT) to a methyl group on unmethylated histone 3 lysine 9 (H3K9) to inhibit pol II transcription, effectively silencing transcription of the gene or TE. The piRNA pathway may also start with a transcript of a protein-coding gene, viral DNA (vDNA), or an invasive viral RNA in the cytoplasm in order to silence the transcript through the ping-pong amplification loop.

The PIWI–piRNA pathway in animals is a conserved pathway that is crucial for genome defense. Its main function is to repress TEs via transcriptional or post-transcriptional silencing mechanisms, thereby maintaining germ-line genomic integrity [32,33,108]. In addition to transposon silencing, piRNAs interact with PIWI proteins to form the piRNA-induced silencing complex (piRISC), which is associated with genome rearrangement, mRNA regulation, epigenetic regulation, spermatogenesis, development, virus defense, and human diseases (Figure 3).

### 4.1. Silencing of Transposable Elements

The first evidence for a small RNA-based regulatory mechanism that could protect against transposon mobilization was noted in repeat-associated small interfering RNAs (rasiRNAs) [4,5,6,12,103]. Since then, abundant TE-related piRNAs have been found in the germ lines of Mollusca, Arthropoda, and Chordata, including fish, dogs, bats, horses, mice, rats, marmosets, and rhesus macaques [11,29,80,106,109,110]. The complexes of piRISC repress transposons via two mechanisms depending on the PIWI protein involved [33]. The cytoplasmic proteins Aubergine (Aub) and Argonaute3 (Ago3) in *Drosophila*, mouse PIWI (Miwi) and Miwi-like protein (Mili) in mice, and silkworm PIWI (Siwi) and Ago3 in silkworms participate in slicer-dependent post-transcriptional gene silencing (PTGS) via the ping-pong cycle [54,103]. In contrast, *Drosophila* PIWI and murine Miwi2 translocate to the nucleus when loaded with piRNAs [54,103,111]. It was found that these molecular mechanisms repress transposons through transcriptional gene silencing (TGS) [15,16,112,113,114,115]. Recent studies have identified novel components of piRNA-mediated TGS; testis expressed 15 (TEX15) and Spen paralogue and orthologue C-terminal domain containing 1 (SPOCD1) might provide a link between piRNA-guided complexes that recognize genomic targets and the molecular machinery that induces DNA methylation and transcriptional repression in mice [116,117,118] and in HP1, histone 3 lysine 9 trimethylation (H3K9me3), small ubiquitin-like modifier (SUMO), and histone deacetylase Rpd3 in *Drosophila* [119,120,121], which would considerably deepen our understanding of PIWI–piRNA-mediated heterochromatin formation at transposon loci. In actual analyses, piRNAs have been found to suppress transposon expression in both somatic and gonadal tissues in *Hydra* [122], *Crassostrea gigas* [27], *Lymnaea stagnalis* [27], and *Pinctada fucata* of Mollusca [123], as well as most Arthropoda [29], which indicates the main role of piRNAs in TE silencing. piRNAs tend to be antisense to transposons and display a preference for a 5 terminal uridine (1U), while piRNAs are primarily in the sense orientation and exhibit a bias for adenosine at position 10 (10A). Moreover, the 5 terminals of sense–antisense piRNA pairs overlap by precisely 10 nt, a relationship termed the ping-pong signature [33,103].

### 4.2. Gene Regulation and Development

In addition to having a role in transposon silencing, piRNAs are also involved in the regulation of cellular genes and pseudogenes, which do not exhibit extensive complementarity to transposons [124,125]. Pachytene piRNA-based RISC containing murine Miwi eliminates mRNA from inactivating cellular processes in preparation for sperm production in elongating spermatids [17]. Miwi–CHIL-seq, gene expression profiling, and reporter-based assays further revealed base-pairing between piRNAs and mRNA targets in mouse testes [85]. Meiotic piRNAs might partially regulate mRNA targets via the ping-pong cycle to enable successful spermatogenesis in mice [126]. RNA interference (RNAi) was used to study a single piRNA (*fem* piRNA) from the silkworm W chromosome, which downregulates z-linked masculinizer (*Masc*) mRNA in response to primary sex determination [127]. In *Drosophila* testes, a Y chromosome-specific piRNA induces sex- and paralog-specific gene regulation of *pirate*, which suggests distinct but related silencing strategies to regulate a conserved protein-coding gene [128]. piRNAs were first demonstrated to engage germ line mRNAs, while tolerating a few mismatches, through perfect pairing at the seed region via miRNA-like pairing rules to regulate gene expression in a model of *C. elegans*, while CLASH analyses and piRNA reporter assays were used to identify piRNA binding sites in detail [89]. The latest research also revealed a piRNA-mediated maternal mRNA decay during the maternal-to-zygotic transition in *Aedes* mosquito and *Drosophila* [98,129]. The role of PIWI–piRNA in gene regulation in development, stem cells, and germ lines has been reviewed previously [130]. Identification of non-transposon piRNA targets is difficult to study in model organisms, and few studies have reported piRNA-mediated gene regulation in non-model animals, although they also possess non-transposon piRNAs [131]. In *P. fucata*, the somatic piRNAs were presumed to regulate endogenous genes by using locked nucleic acid-modified oligonucleotides (LNA antagonists) to silence specific piRNAs in somatic tissues [123]. piRNA-mediated mRNA silencing will provide comprehensive insights into the post-transcriptional regulatory steps in germ-line gene expression in animals.

Recent studies have shown that piRNAs play critical roles in embryonic development in animals, which regulate transposons to maintain genome integrity from parent to offspring [24,47,132]. During the rediscovery of piRNAs from sRNA-seq, abundant piRNAs were also detected in the embryos or early larvae of diverse organisms such as *Drosophila*, cuttlefish, clawed frogs, chickens, and ducks. In Nematostella, piRISCs loaded with mature piRNAs cleave the transcripts derived from TEs as well as protein-coding genes in soma, demonstrating that the roles of piRNAs in transposon repression and gene regulation are likely ancestral features that evolved before the split between Cnidaria and Bilateria [24]. The changes in piRNA composition in different chicken germ line developmental stages and the potential roles of PIWI–piRNA pathways in modulating embryonic stage-dependent TE expression were also investigated [132]. In contrast to most animal species, planarian flatworms also expressed piRNAs in adult stem cells known as neoblasts, where they are required not only for germ line development during the postembryonic stage, but also for tissue renewal, regeneration, and starvation [99,133,134]. In addition, the expression of PIWI proteins and piRNAs in the nervous systems of *C. elegans* [135,136], *Drosophila* [137], *Aplysia* [95], and mice [138,139] may be associated with neurogenesis, learning, and memory. piRNAs also play an essential role in the assembly of telomeric chromatin in the *Drosophila* germ line [140,141]. With the application of sRNA-seq, the roles of piRNAs in the non-canonical functions in animals, especially in embryonic development, nervous system development, and body regeneration, will be progressively discovered.

### 4.3. Epigenetic Regulation

Strong evidence indicates that PIWI–piRNA pathways play a crucial role in epigenetic regulation. piRNAs guide PIWI proteins to specific target sequences in the genome by sequence complementarity to regulate epigenetic processes via histone modification or DNA methylation [142,143,144,145]. Histone modifications are the predominant means by which epigenetic regulation is transmitted from parents to offspring. DNA methylation is another epigenetic silencing marker that is functionally linked to PIWI. Analyses of mouse Mili and Miwi2 indicated that they mediate DNA methylation in the male germ line during embryogenesis [54,146,147]. It may seem that piRNAs can also direct DNA methylation on non-transposon loci, such as the Ras protein-specific guanine nucleotide-releasing factor 1 (*Rasgrf1*) locus in the mouse male germ line to regulate genomic imprinting [148] and the CAMP response element-binding protein 2 (CREB2) promoter in *Aplysia* neurons to influence long-term memory plasticity [95]. Although the molecular mechanism by which piRNAs influence DNA methyltransferases is not clear, the evolutionary conservation of this function is notable. Since piRNAs are involved in epigenetic modifications of gene expression, PIWI–piRNA pathways may play a role in maintaining genome rearrangement and transcriptional or post-transcriptional epigenetic inheritance [38].

### 4.4. Immune Response

Recently, sufficient evidence supporting the involvement of PIWI–piRNA pathways in protection against invading viruses has been found in mosquitoes, although little is known for other insects [149,150]. Eukaryotic genomes contain virus-derived sequences called endogenous viral elements (EVEs), the majority of which are related to retroviruses, which integrate into the host genome for replication [151]. In addition to transposon repression, recent findings support the possibility of an antiviral role for the PIWI–piRNA pathway, suggesting that piRNAs are derived from fragments of RNA viruses [152,153]. Virus-specific piRNAs have been detected in *Drosophila* ovarian somatic sheet (OSS) cell lines, which led to the discovery that the cells were persistently infected with several RNA viruses [154]. However, only virus-derived siRNAs were detected in in vivo studies and they mostly had no effect on viral infection in *Drosophila* mutated for key piRNA pathway proteins [29,155]. In contrast, virus-derived piRNAs, which have ping-pong-specific characteristics, have been reported in a plethora of viral infections, including *Reoviridae*, *Togaviridae*, *Alphaviruses*, and *Bunyavirales* [156]. However, piRNAs against flaviviruses had no ping-pong signatures, except for a slight 10A-bias [149]. An endogenous viral element from a nonretroviral RNA virus produced a set of piRNAs that provided resistance to infection with a cognate virus in the mosquito *Aedes albopictus*, analogous to piRNA-mediated TE silencing in the germ line [157]. Knockdown of key piRNA pathway proteins led to enhanced replication of arboviruses in mosquito cells, suggesting their potential antiviral properties in mosquitoes [158,159]. In addition, metagenomic sequencing data of small RNAs also indicated the presence of an endogenous RNA or DNA virus-derived piRNA expression in divergent animal phyla, including Cnidaria, Echinodermata, and Mollusca [21]. More evidence on endogenous viral element-derived piRNAs supports the hypothesis that they mediate antiviral immunity like clustered regularly interspaced short palindromic repeats (CRISPR) RNAs in prokaryotes [151,160].

### 4.5. Human Diseases (Including Cancer)

Gene expression in cancers is controlled by a variety of regulatory molecules, including small RNAs. Among the three major categories of small RNAs, miRNA profiles in cancers have been extensively characterized, but they are limited in piRNAs. The first report of PIWI expression was in seminomas, a cancer of male germ cells [161]. Since then, ectopic expression of PIWI proteins has been detected in cell lines and tissue samples of a variety of cancers, including those associated with breast, bladder, colorectal, cervical, gastric, liver, and lung cancers [42,43,44,45,162]. A loss-of-function screening for the factors responsible for malignant brain tumors has also demonstrated that PIWI and Aub contribute to tumor growth in *Drosophila* [163]. Furthermore, piRNAs have also been detected in these cancers [43]. Specifically, piRNAs have been found to be differentially expressed in various cancers and cardiovascular diseases [164]. An increasing number of studies have shown that aberrant PIWI and piRNA expression is a signature feature across multiple tumors, which may serve as a novel therapeutic target and biomarker for cancer detection, classification, and therapy [165]. Interestingly, not all piRNAs interact with PIWI proteins in human tumorigenesis. Depletion of piRNA-like-163 (piR-L-163) resulted in accelerated DNA synthesis and G2-M accumulation, as well as increased invasion and cell migration capabilities in human bronchial epithelial cell lines [166]. This occurred through the specific binding of piR-L-163 to phosphorylated ezrin, radixin, and moesin (ERM proteins), which indicates a novel functional role of piRNAs in tumorigenesis. Remarkably, this also reveals another dimension of the functional role of piRNAs in human cancer independent of PIWI proteins. However, the molecular mechanisms and signaling pathways involved in piRNA function in cancers and cardiovascular diseases have not been fully elucidated [43,45,94]. The study of piRNAs will provide new insights into its potential application in clinical diagnoses, prognoses, and therapeutic strategies against human diseases.

## 5. Conclusions

piRNAs are a complex category of small RNAs with non-conserved sequences and functions. They participate in germ-line transposon silencing, genome rearrangement, epigenetic regulation, gene regulation, embryonic development, virus defense, and associated human diseases. Existing research in this area cannot be extended to non-model organisms. The development of sRNA-seq using NGS technologies has dramatically increased the number of newly discovered piRNAs in metazoans over the last decade. In the current review, we presented a common pipeline for piRNA research especially suitable for non-model animals. piRNAs were found to be widely expressed in vertebrate and invertebrate soma and germ lines through the reanalysis of existing sRNA-seq data, suggesting that piRNA function might be broader than previously expected. Further research on piRNA processing is needed to facilitate sRNA-seq analyses in non-model animals.

## Figures and Tables

**Table 1:** Discovery of piRNAs by sRNA-seq in metazoans.

Phylum	Common Name	Species	piRNA Expression	Sources
Nematoda	Nematode	*Caenorhabditis elegans*	Whole organism	[14,18,19,89,97]
Porifera	Sponge	*Amphimedon queenslandica*	Whole organism	[98]
	Sponge	*Halichondria panicea*	Whole organism	[21]
Platyhelminthes	Planarian	*Schmidtea mediterranea*	Whole organism	[20]
	Flatworm	*Macrostomum lignano*	Whole organism	[99]
	Fluke	*Schistosoma japonicum, S. haematobium*, and *S. mansoni*	non-piRNA	sRNAome
	Liver fluke	*Clonorchis sinensis*	non-piRNA	sRNAome
	Tapeworm	*Echinococcus canadensis* and *E. granulosus*	non-piRNA	sRNAome
Annelida	Earthworm	*Lumbricus* and *Amynthas* spps	Bodywall	[21]
	Earthworm	*Eisenia fetida*	Bodywall	sRNAome
Cnidaria	Hydra	*Hydra vulgaris* and *H. magnipapillata*	Whole organism and soma	[22,23]
	Coral	*Acropora muricata, Stylophora pistillata, Montipora capricornis, Montipora foliosa*, and *Pocillopora damicornis*	Polyps	sRNAome
	Sea anemone	*Nematostella vectensis*	Whole organism at different stages	[24]
	Beadlet anenome	*Actinia equina*	Polyps	[21]
	Sea anemone	*Aiptasia pallida*	Polyps	sRNAome
	Jellyfish	*Sanderia malayensis, Rhopilema esculentum*, and *Aurelia aurita*	Appendages, tentacles, rhopalia, oral arms, gonads	[25]
Echinodermata	Starfish	*Asterias rubens*	Tube foot	[21]
	Sea cucumber	*Apostichopus japonicus*	Respiratory tree, tube foot, intestine, body wall	sRNAome
	Sea urchin	*Strongylocentrotus intermedius* and *S. nudus*	Tube foot, larvae	sRNAome
Mollusca	Pacific oyster	*Crassostrea gigas*	Reproductive tract, foot muscle	[27]
	Great pond snail	*Lymnaea stagnalis*	Reproductive tract, foot muscle	[27]
	Snail	*Biomphalaria glabrata*	Adult snail	[100]
	Pearl oyster	*Pinctada fucata*	Adductor, gill, gonad, mantle	[101]
	Pacific abalone	*Haliotis discus hannai*	Adductor muscle	sRNAome
	Common mussel	*Mytilus galloprovincialis*	Hemolymph	sRNAome
	Manila clam	*Ruditapes philippinarum*	Mantle	sRNAome
	Blood clam	*Scapharca broughtonii*	Haemocyte	sRNAome
	Ark shell	*Tegillarca granosa*	Haemocyte	sRNAome
	Cuttlefish	*Sepiella japonica*	Larvae	sRNAome
	Periwinkle	*Littorina littorea*	non-piRNA	sRNAome
	Sea snail	*Rapana venosa*	non-piRNA	sRNAome
	Garden snail	*Helix lucorum*	non-piRNA	sRNAome
	Pearl mussel	*Hyriopsis cumingii*	non-piRNA	sRNAome
Crustacea	Mud crab	*Scylla paramamosain*	Ovary, testis	[28]
	Swimming crab	*Portunus trituberculatus*	Ovary, testis	sRNAome
	Black tiger shrimp	*Penaeus monodon*	Ovary	sRNAome
Arthropoda	Fruitfly	*Drosophila melanogaster* and *D. virilis*	Germline, thorax, embryo	[29]
	Fruitfly	*D. willistoni, D. simulans, D. sechellia, D. pseudoobscura, D. persimilis, D. mojavensis, D. grimshawi, D. erecta, D. ananassae*, and *D. yakuba*	Germline, thorax, head, embryo	sRNAome
	Housefly	*Musca domestica*	Germline, thorax	[29]
	Pea aphid	*Acyrthosiphon pisum*	Germline, thorax	[29]
	Mosquito	*Aedes aegypti*	Germline, thorax	[29]
	Honey bee	*Apis mellifera*	Germline, thorax	[29]
	Bumble bee	*Bombus terrestris*	Germline, thorax	[29]
	Rootworm	*Diabrotica virgifera*	Germline, thorax	[29]
	Postman butterfly	*Heliconius melpomene*	Germline, thorax	[29]
	Horseshoe crab	*Limulus polyphemus*	Germline, thorax	[29]
	Beetle	*Nicrophorus vespilloides*	Germline, thorax	[29]
	Lygaeid bug	*Oncopeltus fasciatus*	Germline, thorax	[29]
	Spider	*Parasteatoda tepidariorum*	Germline, mesosoma	[29]
	Diamondback moth	*Plutella xylostella*	Germline, thorax	[29]
	Centipede	*Strigamia maritima*	Fat body, nerve chord	[29]
	Red flour beetle	*Tribolium castaneum*	Germline, thorax	[29]
	Noctuid	*Trichoplusia ni*	Germline, thorax	[29]
	Scorpion	*Centruroides sculpturatus*	Germline, prosoma	[29]
Chordata	Amur sturgeon	*Acipenser schrenckii*	Ovary, testis	sRNAome
(Fish)	Elephant shark	*Callorhinchus milii*	Ovary, testis	sRNAome
	Bamboo shark	*Chiloscyllium plagiosum*	Liver	[96]
	Epaulette shark	*Hemiscyllium ocellatum*	Non-piRNA	sRNAome
	Zebrafish	*Danio rerio*	Ovary, testis	[80]
	Medaka	*Oryzias melastigma*	Ovary, testis	sRNAome
	Pufferfish	*Takifugu rubripes*	Ovary, testis	sRNAome
	Nile tilapia	*Oreochromis niloticus*	Ovary, testis	sRNAome
	Rainbow trout	*Oncorhynchus mykiss*	Ovary, testis	sRNAome
	Yellow catfish	*Tachysurus fulvidraco*	Ovary, testis	sRNAome
	Stickleback	*Gasterosteus aculeatus*	Ovary, testis	sRNAome
	Ricefield eel	*Monopterus albus*	Mix of brain, liver, and gonad	sRNAome
(Amphibian)	Clawed frog	*Xenopus tropicalis* and *X. laevis*	Ovary, embryo	[102]
(Reptilia)	Alligator	*Alligator sinensis*	Ovary	sRNAome
	Turtle	*Pelodiscus sinensis*	Ovary, testis	sRNAome
	Tortoise	*Mauremys reevesii*	Ovary, testis	sRNAome
	Lizard	*Anolis carolinensis*	Non-piRNA	sRNAome
(Aves)	Chicken	*Gallus gallus*	Ovary, testis, embryo	sRNAome
	Budgerigar	*Melopsittacus undulatus*	Ovary, testis	sRNAome
	Duck	*Anas platyrhynchos*	Embryo	sRNAome
	Goose	*Anser cygnoides*	Ovary	sRNAome
	Pigeon	*Columba livia*	Ovary	sRNAome
(Mammal)	Bat	*Eptesicus fuscus*	Testis	[103]
	Platypus	*Ornithorhynchus anatinus*	Testis	sRNAome
	House	*Equus caballus*	Testis	[104]
	Sheep	*Ovis aries*	Testis	sRNAome
	Dog	*Canis lupus familiaris*	Testis	sRNAome
	Rabbit	*Oryctolagus cuniculus*	Testis, ovary (sRNAome)	[105]
	Cow	*Bos taurus*	Testis	sRNAome
	Pig	*Sus scrofa*	Testis	sRNAome
	Mouse	*Mus musculus*	Testis	[105]
	Rat	*Rattus norvegicus*	Testis	sRNAome
	Opossum	*Monodelphis domestica*	Testis	sRNAome
	Macaque	*Macaca mulatta*	Testis	sRNAome
	Machin	*Macaca fascicularis*	Ovary	[31]
	Marmoset	*Callithrix jacchus*	Testis	[106]
	Human	*Homo sapiens*	Testis	[31,107]

The sRNAome indicated that the piRNAs were discovered from the sRNA-seq data, which were used for the detection of miRNAs but not piRNAs. The datasets did not include all published sRNA-seq data from specific animals or all known animals. In each animal taxon, several representative species were selected for piRNA rediscovery to evaluate the type and quantity of piRNA species during the animal evolution process. The data sources for sRNA-seq are shown in Supplementary Table S1.

## Data Availability

Not applicable.

## Supplementary Materials

The following are available online at www.mdpi.com/article/10.3390/ijms222011166/s1.

## Abbreviations

CLIP-seq: crosslinking immunoprecipitation sequencing; circRNA: circular RNA; lncRNA: long non-coding RNA; NGS: next-generation sequencing; piRNA: PIWI-interacting RNA; PTGS: post-transcriptional gene silencing; qRT-PCR: quantitative reverse transcription-polymerase chain reaction; rasiRNA: repeat-associated small interfering RNA; RIP-seq: RNA immunoprecipitation sequencing; RISC: RNA-induced silencing complex; rRNA: ribosomal RNA; siRNA: small interfering RNA; sncRNA: small non-coding RNA; snoRNA: small nucleolar RNA; sRNA-seq: small RNA sequencing; TE: transposon element; TGS: transcriptional gene silencing; tRNA: transfer RNA.
